# Smaller grafts do not imply early recurrence in recipients transplanted for hepatocellular carcinoma: A Chinese experience

**DOI:** 10.1038/srep26487

**Published:** 2016-05-26

**Authors:** Zhenhua Hu, Xun Zhong, Jie Zhou, Jie Xiang, Zhiwei Li, Min Zhang, Jian Wu, Wenshi Jiang, Shusen Zheng

**Affiliations:** 1 Division of Hepatobiliary and Pancreatic Surgery, Department of Surgery First Affiliated Hospital, School of Medicine, Zhejiang University; Key Laboratory of Combined Multi-organ Transplantation, Ministry of Public Health; Key Laboratory of Organ Transplantation, Zhejiang Province, Hangzhou 310003, China; 2 State Key Laboratory for Diagnosis and Treatment of Infectious Diseases, Collaborative Innovation Center for Diagnosis and Treatment of Infectious Diseases, The First Affiliated Hospital, College of Medicine, Zhejiang University, 310003 Hangzhou, China; 3 China Liver Transplant Registry, Hong Kong, 999077, China

## Abstract

Liver graft size has long been a critical issue in adult-to-adult living donor liver transplantation (LDLT). We analyzed China Liver Transplant Registry data (January 2007–December 2009), identifying 295 patients who underwent LDLT for hepatocellular carcinoma (HCC). The recipients were divided into two groups: A, graft-to-recipient body weight ratio (GRWR) ≤ 0.8% (n = 56); B, GRWR > 0.8% (n = 239). We evaluated donor, recipient, and operative factors and analyzed survival outcome and the risk factors affecting overall and recurrence survival. As a result, the overall survival rates of group B were significantly higher than that of group A (*p* = 0.009); the corresponding tumor-free survival rates did not differ significantly (*p* = 0.133). The overall survival rates among the 151 recipients who met the Hangzhou criteria did not differ significantly (*p* = 0.953), nor did the corresponding tumor-free survival rates (*p* = 0.893). Multivariate analysis determined that GRWR was a significant risk factor for poor survival but not for early recurrence. In conclusion, small grafts may predict poorer survival outcome but do not indicate earlier HCC recurrence in recipients transplanted for HCC, and survival outcome with smaller grafts is merely acceptable in selected recipients.

Survival outcomes are comparable between recipients transplanted for hepatocellular carcinoma (HCC) and those transplanted for nonmalignant diseases with the prerequisite that tumor loads are within the Milan criteria or the Hangzhou criteria^1^,^2^,^3^. Over the past decades, a worldwide shortage of deceased donors has led to the emergence of living donor liver transplantation (LDLT) as the most favorable option for shortening the waiting time of potential recipients, as up to 30% of recipients may develop contraindications to transplantation during this period^4^. For recipients with HCC, the advantages of LDLT over deceased donor liver transplantation (DDLT) remain controversial. Several studies have agreed on LDLT as the preferred strategy for patients with HCC^5^,^6^. However, others have pointed out that LDLT recipients could have higher rates of tumor recurrence^7^,^8^ despite the possible lower waiting list mortality and comparable survival in the long-term. There are multiple reasons for this. One widely accepted point is the selection bias for more aggressive tumor biology among LDLT recipients who might otherwise drop out of the waiting list because of tumor progression. In addition, small-for-size liver grafts could be a critical factor. After LDLT, the implanted partial graft undergoes a complex regeneration process via hepatocyte hyperplasia and hypertrophy^9^, and with the activation of several significant cell signaling pathways, liver regeneration may promote tumor growth, which several basic research studies have already demonstrated^10^,^11^,^12^, leading to a higher rate of tumor recurrence and even poorer survival. For example, Kiuchi *et al.* investigated 276 LDLT recipients and found that recipients with graft-to-recipient body weight ratios (GRWR) <0.8% had significantly worse graft survival^13^. Consequently, we hypothesized that recipients with HCC receiving a relatively smaller graft may face higher tumor recurrence risk and subsequent poorer survival. Basic and clinical studies for discovering the underlying mechanisms would be required for systematic verification of this hypothesis. In the present study, we focused on the role of donor graft size and attempted to gain insight into the impact of GRWR on tumor recurrence and even survival outcome post-LDLT in 295 recipients with HCC.

## Results

### Donors

We compared the following donor parameters between groups: sex, age, graft type (dual, right lobe, left lobe, and other possible types), hepatitis B virus (HBV) infection, and hepatitis C virus (HCV) infection. These clinical parameters were all comparable (Table 1).

### Recipients

Table 2 lists the recipient characteristics. According to the study design, group A had significantly lower graft weight (GW) and GRWR (*p* < 0.001). Sex, age, preoperative status including pre-transplant degradation, pre-transplant vascular invasion, history of spontaneous bacterial peritonitis, alpha fetoprotein levels, preoperative creatinine, operation time, blood loss, cold ischemia time, intensive care unit (ICU) stay, hospital stay, tumor biological status (number of tumor nodules, diameter of the largest tumor, sum of tumor diameters) were not significantly different. 17 patients (50.00%) in group A met the Hangzhou criteria, as did 134 patients (65.69%) in group B (*p* > 0.05).

### Postoperative complications

Table 3 lists the incidence of the most common postoperative complications. Postoperative complications were not statistically different between the two groups.

### Impact of GRWR on survival

**Figure 1:**
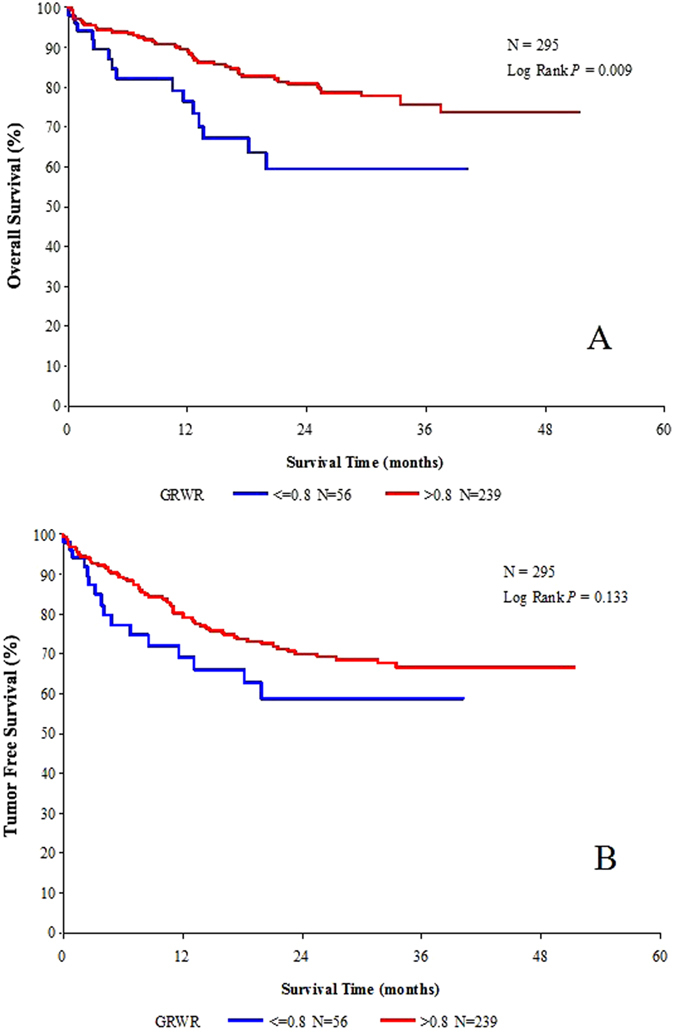
The 1-, 3-year overall survival rates (**A**) and 1-, 3-year tumor-free survival rates (**B**) between group A (GRWR ≤ 0.8) and group B (GRWR > 0.8).

**Figure 2:**
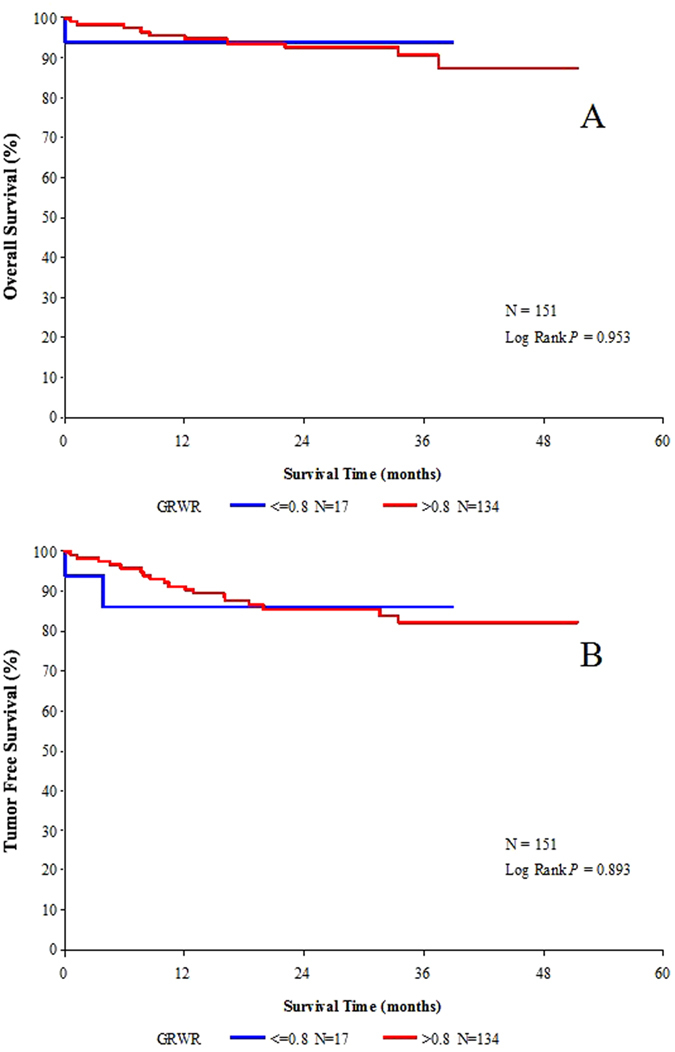
The 1-, 3-year overall survival rates (**A**) and 1-, 3-year tumor-free survival rates (**B**) between group A (GRWR ≤ 0.8) and group B (GRWR > 0.8) of recipients who met the Hangzhou criteria.

Group B (GRWR ≤ 0.8%) had significantly higher overall survival rates than group A (GRWR > 0.8%) (*p* = 0.009) (Fig. 1). However, the corresponding tumor-free survival rates did not differ significantly between the two groups (*p* = 0.133) (Fig. 1). The differences for pre-transplant status, determined based on the number of tumor nodules, size of the largest tumor, preoperative AFP level, and neoadjuvant treatment may have influenced the survival rates significantly. Therefore, we repeated the survival analysis of the 151 recipients who met the Hangzhou criteria. Among these patients, the overall survival rates did not differ significantly between the two groups (*p* = 0.953) (Fig. 2). Similarly, the tumor-free survival rates between the two groups did not differ significantly (*p* = 0.893) (Fig. 2).

There was 3.23% perioperative mortality in the entire cohort, but the two groups were not statistically different (group A: 6.12% versus group B: 2.62%, *p* = 0.198). Repeated survival analysis after excluding cases of perioperative mortality derived a similar outcome to that of the entire cohort (data not shown).

We performed univariate Cox proportional regression analysis to determine risk factors for survival. New Edmondson grading, tumor-node-metastasis (TNM) staging for HCC, vascular invasion, size of the largest tumor, number of tumor nodules, preoperative creatinine, warm ischemia time, and GRWR were associated with survival (data not shown). Multivariate analysis revealed that GRWR, vascular invasion, largest tumor >5 cm, and tumor nodules >4 were associated with decreased survival (Table 4). Univariate analysis excluded GRWR as a predictor of tumor-free survival (data not shown).

## Discussion

The rapidly increasing use of partial grafts in liver transplantation has resulted in more attention being focused on the influence of small graft size on recipient survival outcome. As previously reported, it is recommended to perform graft size selection with GRWR > 0.8% or graft weight/standard liver volume ratio >40% for improving graft survival and for preventing postoperative graft dysfunction^13^,^14^. Nevertheless, Ben-Haim *et al.*^15^ have reported that GRWR as low as 0.6% could be used safely for recipients without liver cirrhosis or with Child A classification. Selzner *et al.*^16^ found no obvious evidence proving that smaller versus larger grafts or full-size deceased donor grafts are associated with poorer outcome. However, the studies mentioned above enrolled LDLT recipients transplanted for multiple reasons and mainly focused on postoperative problems such as small-for-size syndrome and other complications related to small grafts. Moreover, although several clinical studies have demonstrated that, compared to DDLT recipients, LDLT recipients transplanted only for HCC have poorer oncological outcomes^7^,^8^, few studies have concentrated on the effect of smaller grafts on tumor recurrence and the consequent recipient survival outcome, which is exactly what we discuss in the present study.

Theoretically, smaller grafts are associated with higher acute-phase graft injury risk and higher liver regeneration and angiogenesis potential^17^, which creates a better environment for tumor recurrence. Using a well-controlled rat liver transplantation model with liver cancer, Man *et al.*^10^ recently demonstrated that liver graft size and tumor invasiveness were directly associated. Thus, we wondered whether recipients transplanted for HCC may have similar outcomes.

The present study involved 295 LDLT recipients transplanted for HCC. Several significant observations were made after we compared recipients who received grafts with GRWR ≤ 0.8% (group A) versus those who received grafts with GRWR > 0.8% (group B). The 1-, 3-year overall survival rates were significantly higher in group B, while the 1-, 3-year tumor-free survival rates were not statistically different. Repeating the survival analysis of recipients who met the Hangzhou criteria revealed no significant differences in both overall survival and tumor-free survival rates. We found this interesting, and attribute it to the fact that the two groups differed in one aspect, i.e., the 50.0% and 65.7% of recipients who met the Hangzhou criteria in the smaller graft group versus the larger graft group, respectively. Although the difference did not reach statistical significance, it may imply that the group B recipients may be considered highly selected recipients with less aggressive tumor biology and lower tumor burden, thus having better survival outcome. Via univariate and multivariate analysis, we demonstrated that GRWR was a significant risk factor for survival outcome but not for tumor recurrence. The finding was consistent with that of Hwang *et al.*^18^, who performed a single-institution study involving 181 LDLT recipients who had been transplanted for HCC. The present study did not prove the triggering hypothesis. As there is a lack of similar studies, further studies should follow to clarify this inconsistency.

The absence of several important clinical characteristics rendered it difficult to evaluate the impact of GRWR on tumor recurrence and survival outcome more accurately. For instance, Fan *et al.*^19^ reported that, despite similar GRWR values, graft recovery and survival rates were dependent on the inclusion of the middle hepatic vein in right-lobe LDLT; larger grafts were effective in recipients with higher model for end-stage liver disease (MELD) scores^20^; and a recent report by Li *et al.*^21^ found that, in selected recipients with high pre-MELD scores, small grafts were safe, followed by improved intensive care. Therefore, we infer that the differences in surgical options and pre-transplantation MELD scores may be two important factors associated with survival outcome in LDLT recipients. Unfortunately, the present study findings and focus preempt discussion of these factors.

Ours is a retrospective study, and has some limitations. The participants were not randomly assigned to their groups, which inevitablely reflects the limitations of analyzing observational data. The analysis may also have been confounded by the heterogeneous nature of the data from different centers.

In conclusion, we report that among selected LDLT recipients transplanted for HCC, we found no evidence proving that the regeneration of a smaller graft causes early tumor recurrence or poorer survival outcome. With GRWR ≤ 0.8%, overall survival was merely satisfactory. Moreover, GRWR ≤ 0.8% is a predictor of poorer survival but not of early tumor recurrence. The results may aid in addressing the concerns regarding smaller grafts in LDLT recipients transplanted for HCC to some extent, and further studies are warranted to aid in defining the potential accurate lower graft size limits to avoid poorer oncological outcomes in LDLT recipients who are transplanted for HCC.

## Methods

### Participants

**Figure 3:**
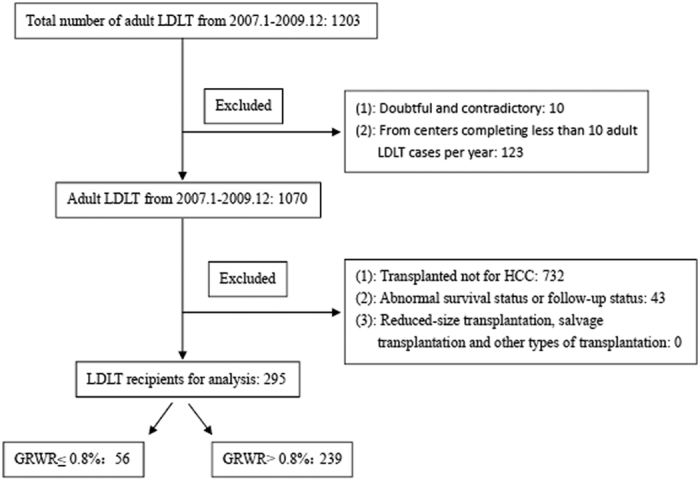
Flowchart of patient enrollment.

Between January 2007 and December 2009, the China Liver Transplant Registry (CLTR) recorded a total 1203 adult LDLT cases from 81 centers across China (Fig. 3). First, participants were selected according to the following criteria: Recipients with HCC, including those with symptomatic and asymptomatic HCC and HCC recurrence following primary liver resection; and recipients who had undergone transcatheter arterial chemoembolization (TACE), radiofrequency ablation (RFA), or other interventional therapies. Then, we excluded the following cases: who had undergone reduced-size, salvage, or other transplantation; who had abnormal survival status; who were absent from data on survival status or follow-up; who were from liver transplantation centers that had completed less than 10 LDLT cases per year. Eventually, 295 LDLT recipients were identified. Their clinical data were collected.

### Procedure

All cases were divided into two groups: A, GRWR ≤ 0.8% (n = 56); B, GRWR > 0.8% (n = 239). The donor and recipient characteristics, major postoperative complications, and perioperative mortality of the two groups were compared. We also calculated and compared the 1-, 3-year overall survival rates and tumor-free survival rates from the operation date. Additionally, we repeated the survival outcome comparisons for recipients who met the Hangzhou criteria.

### Statistical methods

Descriptive statistics are expressed as median (interquartile range). Where appropriate, the chi-square test or Fisher’s test was used for univariate comparisons. Survival outcomes were described using the Kaplan–Meier method. Predictors of survival outcome were identified using univariate and multivariate Cox proportional hazards regression analysis. We considered *p* < 0.05 significant. All statistical analyses were performed by the CLTR using SAS software, version 9.2.

### Ethics statement

We obtained ethical approval from the Zhejiang University Committee of Ethics in Biomedical Research, and the study was approved by the CLTR. Each recipient granted informed consent for the study, and all cases were well evaluated. The protocol conformed to the ethical guidelines of the Declaration of Helsinki as reflected in a priori approval by the institution’s human research committee. This was a hospital-based study.

## Additional Information

**How to cite this article**: Hu, Z. *et al.* Smaller grafts do not imply early recurrence in recipients transplanted for hepatocellular carcinoma: A Chinese experience. *Sci. Rep.* **6**, 26487; doi: 10.1038/srep26487 (2016).

## Figures and Tables

**Table 1:** Donor characteristics.

	GRWR ≤ 0.8%	GRWR > 0.8%	*p*-value
**Donors, n**	**56**	**239**	**–**
Sex (%)			
Male	43 (76.79)	207 (86.61)	0.096
Female	13 (23.31)	32 (13.39)	
Age, years, median (interquartile range)	28.46 (22.95, 41.33)	26.00 (23.33, 36.50)	0.357
Graft type^1^, n (%)			
Dual	0 (0.00)	1 (0.46)	0.585
Right lobe	52 (98.11)	223 (98.62)	
Left lobe	3 (1.89)	4 (0.92)	
Other	0 (0.00)	0 (0.00)	
HBV infection, n (%)			
Yes	0 (0.00)	3 (1.26)	0.589
No	56 (100)	236 (98.74)	
HCV infection^2^, n (%)			
Yes	1 (1.79)	2 (0.84)	0.470
No	55 (98.21)	236 (90.16)	

^1^Twenty-five cases with missing graft type data were excluded.

^2^One case with missing HCV infection data was excluded.

**Table 2:** Recipient characteristics.

	GRWR ≤ 0.8%	GRWR > 0.8%	*p*-value
**Recipients, n**	**56**	**239**	**–**
Sex (%)			
Male	55 (98.21)	216 (90.38)	0.058
Female	1 (1.79)	23 (9.62)	
Age, years, median (interquartile range)	47.35 (42.70, 52.55)	48.60 (43.00, 54.40)	0.714
Body weight, kg, median (interquartile range)	76.00 (68.50, 82.00)	67.00 (60.00, 73.50)	<0.001
GW, g, median (interquartile range)	555.00 (500.00, 607.50)	680.00 (615.00, 743.00)	<0.001
GRWR median (interquartile range)	0.74 (0.69, 0.77)	1.01 (0.90, 1.28)	<0.001
Pre-transplant degradation, n (%)			
Hepatectomy	1 (1.59)	9 (3.05)	0.931
Systemic chemotherapy	0 (0)	3 (1.02)	
radiofrequency ablation (RFA)	2 (3.17)	14 (4.75)	
Transcatheter arterial chemoembolization (TACE)	0 (0)	64 (22.18)	
Percutaneous ethanol injection	11 (19.64)	1 (0.42)	
Combined treatment	2 (3.57)	17 (6.28)	
None	40 (71.43)	187 (61.51)	
Pre-transplant vascular invasion, n (%)	44 (48.57)	184 (76.99)	0.861
History of hepatic encephalopathy	2 (3.57)	14 (5.86)	0.745
History of SBP	0 (0)	3 (1.26)	1.000
AFP level^1^, ng/mL, median (interquartile range)	231.60 (9.70, 1210.00)	107.75 (9.20, 1000.00)	0.156
Preoperative creatinine^2^, μmol/L, median (interquartile range)	65.10 (57.70, 75.60)	67.00 (58.10, 75.00)	0.472
Operation duration^3^, hours, median (interquartile range)	10.80 (9.50, 12.85)	10.30 (8.00, 12.50)	0.489
Blood loss^4^, mL, median (interquartile range)	1800.00 (1000.00, 3000.00)	2000.00 (1000.00, 3000.00)	0.769
Cold ischemia time^5^, hours, median (interquartile range)	0.91 (0.33, 2.50)	1.48 (0.90, 2.13)	0.070
Warm ischemia time^6^, hours, median (interquartile range)	0.00 (0.00, 0.50)	0.00 (0.00, 1.00)	0.017
ICU stay after transplantation^7^, hours, median (interquartile range)	144.00 (82.00, 230.00)	125.00 (72.00, 192.00)	0.088
Hospital stay after transplantation^8^, days, median (interquartile range)	35.00 (28.00, 59.00)	36.00 (27.00, 51.00)	0.545
Length of follow-up, months, median (interquartile range)	11.06 (1.99, 20.59)	23.78 (8.58, 34.80)	<0.001
Tumor nodules^9^, n, median (interquartile range)	1.00 (1.00, 2.50)	1.00 (1.00, 3.00)	0.257
Largest tumor diameter^10^, cm,			
median (interquartile range)	4.00 (2.60, 5.00)	3.50 (2.40, 5.50)	0.701
Sum of tumor diameters^11^, cm, median (interquartile range)	5.00 (2.50, 7.50)	4.10 (2.50, 7.00)	0.580
Hangzhou criteria^12^			
In	17 (50.00)	134 (65.69)	0.086
Out	17 (50.00)	70 (34.41)	

^1^Ten cases with missing AFP data were excluded.

^2^Two cases with missing creatinine data were excluded.

^3^Five cases with missing or abnormal operation time data were excluded.

^4^Ninety-three cases with missing or abnormal blood loss data were excluded.

^5^Thirty-seven cases with missing or abnormal cold ischemia time data were excluded.

^6^Fifty-nine cases with missing or abnormal warm ischemia time data were excluded.

^7^Five cases with missing, logical paradoxes or abnormal ICU time data were excluded.

^8^Fifteen cases with missing, logical paradoxes or abnormal hospital time data were excluded.

^9^Sixty-nine cases with missing nodule or abnormal nodule data were excluded.

^10^Fifty-two cases with missing size or abnormal size data were excluded.

^11^Eighty-nine cases with missing sum of tumor diameter or abnormal sum of diameter data were excluded.

^12^Fifty-seven cases with abnormal tumor feature data were not included in Hangzhou criteria evaluation.

**Table 3:** Postoperative complications.

Postoperative complications	n	GRWR ≤ 0.8	n	GRWR > 0.8	*p*-value
		Incidence (%)		Incidence (%)	
**Recipients, n**	**56**		**239**		**–**
Pleural effusion	20	35.71	96	40.17	0.648
Diabetes mellitus	10	17.86	70	29.29	0.095
Intra-abdominal collection/abscess	13	23.21	79	33.05	0.199
Bacterial infection	8	14.29	43	17.99	0.694
Hypertension	7	12.50	52	21.76	0.139
Biliary complication	9	16.07	53	22.18	0.366
Hyperlipidemia	4	7.14	24	10.04	0.619
Hypercholesterolemia	4	7.14	23	9.62	0.796
Intra-abdominal bleeding	3	4.76	9	3.77	0.705
Vasculitis	4	7.94	12	5.02	0.516
Renal failure	1	1.79	5	2.09	1.000
Chronic injection	0	0	3	1.26	1.000
Pulmonary edema	0	0	1	0.42	0.334
Graft dysfunction	0	0	1	0.42	1.000
Cyclosporin A toxicity	0	0	0	0	–
Graft-versus-host disease	0	0	0	0	–
**Total**	**38**	**67.86**	**171**	**71.55**	**0.625**

**Table 4:** Multivariate analysis results of risk factors for survival after LDLT (n = 295).

Factor*	Group		Reference group	Parameter estimate	*p*	Hazard ratio	Hazard ratio 95% confidence interval		*p*
Age, years	18–29	vs.	≥65	−12.149	0.987	0.000	0.000	–	0.199
	30–39			−0.038	0.974	0.963	0.096	9.656	
	40–49			1.233	0.238	3.431	0.444	26.547	
	50–64			0.864	0.406	2.371	0.310	18.147	
Sex	Male	vs	Female	0.531	0.376	1.701	0.525	5.514	0.375
Vascular invasion	Yes	vs	No	1.107	<0.001	3.024	1.770	5.166	<0.001
Transplant year	2007	vs	2009	−0.214	0.593	0.807	0.368	1.770	0.677
	2008			0.047	0.898	1.048	0.510	2.153	
GRWR	≤0.8%	vs	>0.8%	0.773	0.014	2.166	1.173	4.001	0.013

^*^Adjusted for transplant year, recipient sex, recipient age, and GRWR. The whole model is significant (*p* < 0.001).
